# Predictors of cervical cancer screening practice among HIV positive women attending adult anti-retroviral treatment clinics in Bishoftu town, Ethiopia: the application of a health belief model

**DOI:** 10.1186/s12885-019-6171-6

**Published:** 2019-10-23

**Authors:** Kalkidan Solomon, Mulugeta Tamire, Mirgissa Kaba

**Affiliations:** Department of Preventive Medicine, School of Public Health, College of Health Sciences, Addis Ababa University, Addis Ababa, Ethiopia

**Keywords:** Cervical cancer, Screening, HIV/AIDS, Health belief model

## Abstract

**Background:**

Cervical cancer is a global public health problem and the second most common cancer causing morbidity and mortality in Ethiopia. Few available evidences revealed that despite distribution and severity of cervical cancer among HIV-positive women and the ease with which it can be prevented, cervical cancer screening practice in Ethiopia among them is considerably low. Thus, this study aims to assess predictors of cervical cancer screening practice among HIV-positive women by applying health belief model concepts.

**Methods:**

Facility based cross-sectional study was conducted in Bishoftu. Data was collected from 475 women who visit the health facilities for anti-retroviral services using interviewer-administered questionnaires. Champion’s revised Health Belief Model sub-scales were used as data collection tools containing sources of information, knowledge, perception on cervical cancer screening and cervical cancer screening practice as variables. Frequencies, percentage, mean and standard deviation were used to describe findings. Multi-variable logistic regression and 95% confidence intervals were considered to identify predictors of cervical cancer screening practice by controlling possible confounders.

**Results:**

Cervical cancer screening practice among HIV-positive women in this study was 25%. Health proffesionals were the main sources of information about cervical cancer and its screening. There was a difference between the ‘ever’ and ‘never’ screened groups in mean scores of their perceived severity, perceived benefit, perceived barrier, perceived self-efficacy, perceived threat and net-benefit towards screening (*P* < 0.05). Perceived self-efficacy (AOR 1.24, 95%CI 1.13–1.37), perceived threat (AOR 1.08, 95%CI 1.05–1.12) and perceived net-benefit (AOR 1.18, 95% CI 1.12, 1.24) were the predictors of cervical cancer screening practice.

**Conclusions:**

Cervical cancer screening practice in this study was lower than that of the recommended coverage of the target group by the national guideline (80%). This finding has an important implication for public health intervention aimed at cervical cancer prevention. Morever, womens’ perceptions on cervical cancer screening had a significant influence on the utilization of cervical cancer screening service. Therefore, educational programmes geared towards severity of the case, availability of screeningand helpfulness of being screened can significantly improve the uptake of cervical cancer screening.

## Background

Cervical Cancer (CC) is malignancy of the cervix caused by the presence of human papilloma virus (HPV) which interferes with the normal functioning of cells that will result in a distinct change in the epithelial cells of transformation zone of the cervix [1]. Cervical cancer is an alarmung public health problem worldwide [2–4]. In developing countries, it is the second most commonly diagnosed cancer after breast cancer and the third leading cause of cancer death after breast and lung cancers [3]. In Sub-Saharan Africa, it is the most common cause of female cancer-related deaths [5]. In Ethiopia, cervical cancer is the second most frequently diagnosed cancer and the leading cause of cancer mortality among women aged 15 to 44 years [6]. There were 29 million women aged 15 years and older at risk of cervical cancer in the countryin the year 2010 [7]. Every year 7600 women are diagnosed with cervical cancer and approximately 5000 die from the disease [7, 8].

According to Ehiopian demographic health survey (EDHS) 2016 report, HIV/AIDS prevalence among women aged 15 to 49 years was 1.2% [9]. In Ethiopia 534,000 women age 15 year and above are living with HIV. These are among the most vulnerable to cervical cancer since their risk of pre-cancerous lesions are 10 times greater and are more likely to progress to invasive cervical cancer compared with uninfected women [10, 11]. CC has bimodal distribution in relation to age, one at 30 years and other at 60 years [12]. These two age groups generally become symptomatic to cervical lesion while those women who are HIV-positive are symptomatic to cervical cancer irrespective of the age distribution [13, 14].

According to world health organization (WHO) guideline, every sexually active woman aged 30–49 years should undergo cervical cancer screening at least every 5 years. However, sexually active and HIV-positive womenare suggested to be screened every 3 years regardless of their age [15]. Ethiopia adoptedtheWHO recommendation in 2015 and recommended HIV positive women to start screening at HIV diagnosis, regardless of age and re-screen every 5 years [11]. The government of Ethiopia has given more emphasis on programs focusing on the early detection of cervical cancer. Several advocacy efforts were made by different stakeholders such as academia, professionals, media and development partners to combat cervival cancer [6].

In spite of the high prevelnce and severity of the problem among at risk women in low income countries and the fact that it is the only gynaecologic cancer which can be prevented and treated through early screening and follow-up, cervical cancer screening practice in low income countries among HIV-positive women is considerably low [16, 17]. In Ethiopia, cervical cancer screening service utilization among HIV-positive women is much lower than the national recommended coverage of 80% [7, 11, 18–21]. The accessibility of effective cervical screening programs will only be useful if utilized by the target population, since the goal of the national government in introducing this program is far from being achieved as relatively very few women have actually done cervical cancer screening [11]. In Ethiopia, cervical cancer screening is offered routinely at out-patient, ART, and maternal and child health departments. Of the numbers attending the departments, less than 25% of eligible women have actually done CC screening [18, 22–25]. Thus to maximize the uptake to reach more vulnerable women including those who are HIV-positive, it is necessary to know those factors which affect HIV-positive women’s behavior to get screened.

A health belief model (HBM) is a model that assumes the best predictor of a behavior is an individual’sperception [26]. However, studies which focus on a perception towards cervical cancer and its screening are not that frequent in Ethiopia. The paucity of studies in this regard seems to create an information gap among both study subjects and stakeholders. Therefore, this study aimed to assess predictors of cervical cancer screening practice among HIV positive women based on the perspective of a Health Belief Model.

The study addressed modifiable factors for poor screening practice, women’s perception, which affects the screening practice and gaps between the women’sknowledge on cervical cancer and actual practice. Further, it helps policy-makers and non-governmental organizations (NGO) working on cancer design evidence-based cervical cancer control and prevention programs among HIV-positive women and provide a convenient programmatic approach to address factors affecting cervical cancer screening practice.

## Methods

### Study design and area

Facility based cross-sectional study was conducted in Bishoftu town, East Shoa, Ethiopia, from January 15th to April 5th, 2018. Bishoftu is located 47 km south-east of Addis Ababa, the capital city of Ethiopia. According to the 2007 census projection, the total population of the town was estimated to be around 119,845 in 2016 [27, 28]. Currently in the town, 23,410 are recorded to live wih HIV/AIDS, 5601 of these are women; of the total adult HIV-positive, 4164 are registered and actively followed in the ART clinics, of them, 2827 are women. Anti-retroviral treatment (ART) services are given in the Bishoftu hospital and Bishoftu health center. See and treat approach by using visual inspection with acestic acid (VIA) and cryotherapy as cervical cancer screening modality is available in Bishoftu hospital which is provided twice a week with two trained screening service providers.

### Study population

All HIV-positive women registered for and on ART services aged 18 years and above atART clinics in Bishoftu townwere eligible for the study. Among these, HIV-positive women with confirmed cancer of the cervix and those who developed ART-Anti Tuberculosis (TB) drug reaction were excluded from the study.

### Sample size determination

The sample size was calculated using a single population proportion formulawith Open-epi software version 2.3.The following assumptions were made:confidence level of 95% (za/2 = 1.96), 4% margin of error, the proportion (p) of cervical cancer screening in women living with HIV/AIDS (24%) from a previous study conducted in Ethiopia which gave maximum sample size, [21] and 10% non-response rate were taken to determine sample size. Accordingly the sample size was determined at 482.

### Sampling procedures

The sample size was proportionally allocated to Bishoftu General Hospital and BishoftuHealth Centre basedon 3 months ART clients’ flow preceeding actual data collection. Out of 2827 HIV-positive women visiting ART clinics in Bishoftu town, two women who had confirmed cases of cervical cancer and five women who developed ART/Anti TB drug reaction were excluded before proportional allocation of the study participants to each health institution. Accordingly 354 samples were allocated to the hospital and 118 to the health centre. Finally, the study participants were selected from each health facillity using simple random sampling technique, by computer-aided random selection using the participants ART register identification number as sampling frame after filtering those who are excluded.

### Measurements

Data was collected using an interviewer administered structured questionnaire that measuressocio-demographic characteristics, knowledge, Health Belief Model constructs and cervical cancer screening practice Additional file 1. The structured questionnaire was adapted from Champion’s revised Health Belief Model Scale (CHBMS) developed in 1993 [29, 30] and other studies [18, 21, 31–33].

Each question for HBM constructs except cues to action was scored using a 5-point Likert scale ranging from strongly agree (5) to strongly disagree (1). Perceived susceptibility, which was defined as the views of HIV-positive women regarding their risk of having cervical cancer, has a total of 5 items scored from 5 to 25. Perceived severity of cervical cancer which was a subjective assessment of how serious cervical cancer was viewed by these HIV-positive women has 9 items scored from 9 to 45. Perceived benefit which was viewed as the perception that cervical cancer screening will result in early detection, delay progression and subsequently lead to decrease mortality due to cervical cancer has 6 items scored from 6 to 30 [29, 34]. Perceived barrier, which was viewed as obstacles preventing participation in the available cervical cancer screening programmes has 15 items scored from 15 to 75. Perceived self-efficacy, viewed as the conviction that HIV-positive women can successfully execute the behavior required to practice cervical cancer screening, consisted of 5 items scored from 5 to 25. We used the sum score of perceived susceptibility plus perceived severity to measure perceived threat and the sum score of perceived benefit minus perceived barrier for perceived net benefit [29, 34]. ‘Cues for action’ which was viewed as trigger actions to practice cervical cancer screening, consisted of 4 items with ‘yes or no’answers and rated one for no and two for yes responses. Knowledge towards cervical cancer and cervical cancer screening contain a total of 14 items with ‘yes’ or ‘no’ answers with one point score for each correct response and zero if incorrect. The categorical dependent variable rated ‘yes’ or ‘no’ was whether a woman had ever had cervical cancer screening.

The questionnaire was prepared in English and translated by a language expert from the English version to the Amharic language then to Afaan Oromo before data collection. We pre-tested the tool on 10% of the total sample size in Adama Hospital 2 weeks prior to the actual data collection and modifications were made from the result of the pre-test.

Data were collected by four experienced ART service providers after 1 day of training on the objective, methodologies, tool and data collection techniques of the study. The interview was conducted in ART counseling rooms to create confidentiality within a secure environment. Each interview took an average of 40 min. The supervisor provided hands on supervision to ensure data quality.

Data was intensively cleaned and negatively stated items were reversed before analysis. The psychometric properties of the CHBMS were tested through construct validity and internal consistency in order to addressmeasurement variability [26]. Confirmatory factor analysis (CFA) showed all the factor loadings were more than 0.4except one item from perceived severity and three from perceived barrier. The average variance extracted (AVE) for all the constructs were more than 0.5. The Cronbach alpha > 0.7 [35] confirmed internal consistency of the dimension, which was 0.90 for perceived susceptibility, 0.87 for perceived severity, 0.85 for perceived barrier, 0.77 for perceived self-efficacy and 0.69 for perceived benefit and cues to action.

Normality of the data, homogeneity of variance, multi-collinearty and interaction were checked before running any kind of analysis. Existence of multi-collinearty between each of the constructs of Health Belief Model including knowledge, was checked and there were no multi-collinearty among them (VIF < 10). Existence of interaction among perceived susceptibility and perceived severity (*p* = 0.20), perceived benefit and perceived barrier (*p* = 0.38), perceived threat and net-benefit (*p* = 0.22), perceived susceptibility and cues to action (*p* = 0.06) were checked and there were no effect modification between them.

### Data managementand analysis

The responses in the completed questionnaire were coded and entered into Epi-data version 4.2.0.0 and exported to Statistical Packages for Social Sciences (SPSS) version 23 for analysis.

All analysis has compared HIV-positive women who had ever had cervical cancer screening with those who had never had cervical cancer screening. First the descriptive statistics was used to describe frequency distribution, proportion, measures of central tendency and dispersion. Perception of participants were measured using HBM constructs and treated as continuous variables [36].

Mean and standard deviation were generated for each of HBM constructs and knowledge items. For all constructs of HBM, the responses were summed up and a total sum score of their responses was computed with possible values ranging from minimum to maximum value.

Independent sample t-tests were used to determine whether mean differences existed for perceived susceptibility, perceived seriousness and perceived threat towards cervical cancer, perceived barriers, perceived benefits, perceived self-efficacy, perceived net-benefit and cues to action towards cervical cancer screening, between women who had ever screened for cervical cancer and women had never screened for cervical cancer. Crude odds ratios and 95% confidence intervals were generated from binary logistic regression as measures of associations for each socio-demographic characterstics, HIV/AIDS related variables, knowledge and for aggregate score of each health belief model constructs with cervical cancer screening practice. A multi-variable logistic regression was used to identify predictors of cervical cancer screening by controlling the possible confounder. Based on *P*-values less than 0.25 in bivariate analysis, [37] consideration of multi-collinearity, clinical significance and maximum number of variables which is reasonable to enter into the model, [38] 12 variables were included in multi-variable logistic regression analysis. Statistical significance for the multi-variable logistic regression analysis was set at *p* ≤ 0.05. The Hosmer-Lemeshow Goodness of Fit tests were used to check whether the model adequately fits the data in this study.

## Results

### Socio-demographic and clinical characteristics of study participants

From 482 women identified as eligible for the study, four of them missed their ART appointment; three of them were excluded because of incomplete data leaving a total of 475 HIV-positive women participated and completed the interview with 98.5% response rate. The age of the respondent’s ranged from18 to 67 years with a mean age of 36.20 ± (SD 10.30) and median age of 34.00. Half the participants were followers ofthe Orthodoxreligion. Majority of the respondents298 (62.70%) were married, 228(48.00%) had one child and 30(6.30%) were grand Parous. Two-thirds of the participants 319(67.20%) did not attend formal education, approximately half, 252(53.10%) were government employees and 138(29.10%) reported to earn 800 Ethiopian birr per month (Table 1). The majority of the participants 236 (49.70%) were diagnosed as HIV-positive before 4 years ago, about half 240 (50.50%) started ART before 4 years and most of the participants 195 (41.50%) were in WHO disease stage one (Table 1).

**Table 1:** Socio-demographic and clinical characteristics of the study participants, Bishoftu, Ethiopia, 2018

Variables	Frequency(*n* = 475)	Percentage
Religion		
Orthodox	235	49.50
Muslim	171	36.00
Protestant	51	10.70
Catholic	18	3.80
Ethnicity		
Oromo	215	45.30
Amhara	174	36.60
Tigrie	57	12.00
Gurage	29	6.10
Marital status		
Married	298	62.70
Divorced	80	16. 90
Widowed	57	12.00
Single	40	8.40
Parity		
None(nullipara)	103	21.70
One	228	48.00
Two-four(multi para)	114	24.00
>four(grand para)	30	6.30
Educational level of the participant		
Illitrate	319	67.20
Litrate	156	32.80
Occupational status of the participant		
Unemployed	112	23.60
Gov’t employed	252	53.00
Self-employed	111	23.40
Educational level of the participants’ husband (*n* = 298)		
Illiterate	194	65.10
Literate	104	34.90
Occupational status of participants’ husband (*n* = 298)		
Unemployed	7	2.35
Employed	240	80.54
Self-employed	51	17.11
Monthly income in ETB (*n* = 475)		
1st quartile(≤800)	138	29.00
2nd quartile(801–1100)	100	21.10
3rd quartile(1101–200)	122	25.70
4th quartile(> 2000)	115	24.20
WHO clinical stage of HIV/AIDS		
1	195	41.10
2	191	40.20
3	51	10.70
4	38	8.00
Duration of HIV infection		
< 4 year	236	49.70
4–8 year	143	30.10
> 8 year	96	20.20
Duration on ART		
< 4 year	240	50.50
4–8 year	148	31.20
> 8 year	87	18.30

*ETB* Ethiopian birr (1ETB~27US$)

### Source of information and knowledge of cervical cancer and cervical cancer screening

Four hundred and twenty one (421) (88.60%) respondents had heard about cervical cancer. Of these, 342(81.20%) heard from health care providers and 67(15.90%) via the media. Of these total respondents who have ever heard about CC, 398(94.50%) heard about the presence of cancer screening, of these 248(62.30%) heard from healthcare providers and 138(34.70%) via the media (Table 2). Knowledge of cervical cancer and its screening was analyzed as a continuous variable with observed values ranging from 20 to 34 with the mean knowledge score of 25.93 (SD ± 2.31) (Table 2).

**Table 2:** Source of information and knowledge of cervical cancer and cervical cancer screening of respondents, Bishoftu, Ethiopia, 2018

Variables	Frequency			Precent	
Heard about cervical cancer (*n* = 475)					
Yes	421			88.60	
No	54			11.40	
Source of info about cervical cancer for the last time (*n* = 421)					
Health care providers	342			81.20	
Media(print and non-print)	67			15.90	
Close relatives(family/friends)	12			2.90	
Heard about Cervical cancer screening (*n* = 421)					
Yes	398			94.50	
No	23			5.50	
Source of info about Cervical cancer screening for the last time(*n* = 398)					
Health care providers	248			62.30	
Media(print and non-print)	138			34.70	
Close relatives(family/friends)	12			3.00	
	Scale	Minimum	Maximum	Mean	SD
Knowledge^a^	0–40	20	34	25.93	2.31

^a^Continuous variable, *SD* Standard deviation

### Cervical cancer screening practice among HIV positive women

A quarter of respondents, 118 (24.80, 95%CI 21.00–28.00%) reported to have ever screened for cervical cancer. Of these 98 (83.00%) actually had the screening within the past year, 76 (64.40%) were advised by a health care provider and 104 (88.10%) had been screened after diagnosed for HIV/AIDS (Table 3).

**Table 3:** Cervical cancer screening practice among HIV positive women, Bishoftu, East Shoa, Ethiopia, 2018

Variables	Frequency	Percentage
Cervical cancer screening (*n* = 475)		
Ever screened	118	24.80
Never screened	357	75.20
Reason for screening (*n* = 118)		
Healthcare provider advice.	76	64.40
Being sick (Illness)	39	33.10
Relatives (family/friends) advice	3	2.50
Screened after HIV diagnosis		
Yes	104	88.10
No	14	11.90
Frequency of screening		
Once	68	57.60
Twice	50	42.40
Last screening time		
Just a year	98	83.00
Two years before	8	6.80
Three years before	12	10.20

Women who had never screened (357) were asked their reasons and choosing more than one reason was possible. The most identified reason for not considering cervical cancer screening were fear of positive result (28.00%), feeling of a woman as being healthy (20.00%) and partner attitude (15.00%).

### Comparison of HIV positive women’s perceptions among those ever and never screened for cervical cancer

Perception of participants was measured using Health Belief Model constructs and were analyzed as a continuous variable with mean score 18.48 (SD ± 4.50) for perceived susceptibility, 33.65 (SD ± 8.59) for severity, 23.75 (SD ± 3.63) for benefit, 56.43 (SD ± 6.17) for barrier, 20.23 (SD ± 3.06) for self-efficacy and 6.68(SD ± 1.19) for cues to action (Table 4).

**Table 4:** Perception of HIV positive women visiting ART clinic in Bishoftu town, East Shoa, Ethiopia, 2018 (*n* = 475)

S.no	Constructs	Scale range	25th percentile	50th percentile	75th percentile	Mean	SD
1	Perceived susceptibility^a^	5–25	15	20	21	18.48	4.50
2	Perceived severity^a^	9–45	31	36	39	33.65	8.59
3	Perceived benefit^a^	6–30	23	24	26	23.75	3.63
4	Perceived barrier^a^	15–75	53	57	61	56.43	6.17
5	Perceived self-efficacy^a^	5–25	10	20	22	20.23	3.06
6	Cues for action^a^	4–8	4	5	6	6.68	1.19

^a^indicates continuous variable, SD: standard deviation

There was significant difference between ever and never screened in terms of perceived severity, perceived benefit, perceived barrier, perceived self-efficacy, perceived threat and net-benefit (*P* < 0.05) (Table 5). Women who had ever screened for cervical cancer had significantly higher perceived severity (t = 2.316; *P* = 0.021), higher perceived benefit (t = 3.295; *P* = 0.001), higher perceived self-efficacy (t = 3.470; *P* = 0.001), higher perceived threat (t = 2.647; *p* = 0.008) and higher perceived net benefit (t = 4.570; *p* = 0.001). Women who had ever screened for cervical cancer had lower perceived barrier (t = − 2.303; *P* = 0.022). There was no significant mean difference for perceived susceptibility (t = 1.358; *P* = 0.175) and cues for action (t = − 1.261; *p* = 0.208) between both groups (Table 5).

**Table 5:** Comparison of perception among ever and never screened HIV-positive women for cervical cancer

S.no.	Predictor variables	Ever Screened		Never Screened		*t*-value	*P*-value	95% CI
		Mean	SD	Mean	SD			
1	Perceived susceptibility	18.97	4.36	18.32	4.50	1.358	0.175	−0.290, 1.589
2	Perceived severity	35.23	7.50	33.13	8.80	2.316	0.021	0.318, 3.886*
3	Perceived benefit	24.69	3.65	23.43	3.57	3.295	0.001	0.507, 2.008*
4	Perceived barrier	55.30	5.80	56.80	6.20	−2.303	0.022	−2.787, −0.220*
5	Perceived self-efficacy	21.07	2.85	19.96	3.08	3.470	0.001	0.483, 1.747*
6	Perceived threat	54.21	9.27	51.45	9.95	2.647	0.008	0.709, 4.795*
7	Net benefit	−30.61	6.36	−33.37	5.45	4.570	0.000	1.574, 3.950*
8	Cues for action	6.56	1.27	6.79	1.17	−1.261	0.208	−0.410, 0.089

*Indicates significant mean difference (*P* < 0.05)

### Predictors of cervical cancer screening practice

Among socio-demographic variables only participant occupation was significant in explaining cervical cancer screening practice. Furthermore, among constructs of Health Belief Model, perceived self-efficacy, perceived threat and net benefit were independent predictors of cervical cancer screening practice (Table 6).

**Table 6:** Predictors of cervical cancer screening practice among HIV positive women, Bishoftu, Ethiopia, 2018

Variables	COR(95%CI)	*P*-value	AOR(95%CI)	*P*-value
Marital status				
Single	1		1	
Married	2.531 (1.027–6.237)	0.044	1.415 (0.372–5.380)	0.610
Divorced	0.903 (0.308–2.650)	0.853	0.625 (0.138–2.837)	0.543
Windowed	1.062 (0.346–3.265)	0.916	1.248 (0.277–5.630)	0.773
Parity				
None(nullipara)	1		1	
One	2.734 (1.520–4.918)	0.001	1.623 (0.607–4.335)	0.334
2–4(multi para)	0.708 (0.330–1.520)	0.376	0.687 (0.246–1.916)	0.473
> 4(grand para)	1.540 (0.570–4.157)	0.394	1.077 (0.288–4.031)	0.912
Educational level of the participant				
Illiterate	1		1	
Literate	0.435 (0.265–0.715)	0.001	0.978 (0.507–1.888)	0.948
Occupational status of the participant				
Unemployed	1		1	
Gov’t employed	4.840 (2.467–9.502)	0.000	5.505 (2.628–11.532)	0.020*
Self employed	2.018 (0.917–4.431)	0.081	3.047 (1.298–7.151)	0.018*
Monthly income				
1st quartile	1		1	
2nd quartile	1.128 (0.639–1.990)	0.678	1.059 (0.510–2.196)	0.879
3rd quartile	0.896 (0.516–1.558)	0.698	1.251 (0.619–2.528)	0.532
4th quartile	0.521 (0.281–0.966)	0.039	0.650 (0.312–1.355)	0.250
Time of HIV diagnosis in year				
< 4	1		1	
4–8	0.433 (0.258–0.726)	0.002	0.498 (0.126–1.969)	0.320
> 8	0.530 (0.299–0.938)	0.029	0.734 (0.190–2.827)	0.650
Duration of follow up for ART				
< 4	1		1	
4–8	0.402 (0.240–0.672)	0.001	1.528 (0.813–2.869)	0.188
> 8	0.408 (0.255–0.858)	0.014	1.410 (0.540–3.681)	0.483
Knowledge^a^	0.961 (0.923–1.001)	0.054	1.081 (0.345–3.390)	0.894
Self-efficacy^a^	1.156 (1.063–1.253)	0.001	1.242 (1.128–1.368)	0.000*
Threat^a^	1.031 (1.008–1.055)	0.009	1.086 (1.052–1.120)	0.000*
Net- benefit^a^	1.084 (1.046–1.124)	0.001	1.181 (1.122–1.243)	0.000*
Cues to action^a^	0.895 (0.752–1.064)	0.208	1.009 (0.816–1.249)	0.932

^a^continuous variables, *indicates predictor of cervical cancer screening practice (*p* < 0.05)

Keeping all other factors constant, the odds of having cervical cancer screening were about 6times higher for those participants who were government employee (AOR 5.505, 95% CI 2.628, 11.532) and 3 times higher for those participants who were self-employed (AOR 3.047, 95%CI 1.298, 7.151) than those who were unemployed (Table 6).

After holding all other variables constant, per a unit increases in total score of perceived self-efficacy towards cervical cancer screening increased the odds of practicing cervical cancer screening by 24.20%, (AOR 1.242,95% CI 1.128, 1.368), whereas a unit increase in total score of perceived threat increased the odds of practicing cervical cancer screening by 8.60% (AOR 1.086, 95% CI 1.052, 1.120). The other variable independently associated with cervical cancer screening practice was net benefit, a unit increase in total score of perceived net benefit increased the odds of practicing cervical cancer screening by 18.10% (AOR 1.181, 95% CI 1.122, 1.243) (Table 6). For this study the model adequately fits the data (*p* = 0.433).

## Discussion

This facility-based study showed that out of 475 study participants, only a quarter of them were ever tested for cervical cancer. This cervical cancer screening practice is too low and less than the National Ministry of Health goal of screening at least 80% or more of eligible women for cervical cancer [6]. The level of screening in this study was comparable with the findings of the study conducted in Gonder, Ethiopia, which showed that the magnitude of ever screening for cervical cancer among HIV-positive women was 24% [21]. On the other hand, the finding of this present study was higher compared with the study among patients living with HIV/AIDS in Addis Ababa, Ethiopia (11.50%) [18] and a study done in Zimbabwe (9%) [39]. The higher uptake of screening service in this study could be explained by the improved expansion and access of screening centres, for instance diferent types of screening have become a routine procedure and part of the standard care for women who are diagnosed as HIV-positive, [11] the enhanced nation-wide advocacy, media concern, community sensitization and awareness creation about the cervical cancer screening that has been put into effect time-to-time [40].

Despite the recent effort to screen all HIV-positive women who are on ART and who have no history of being screened, the proportion of women screened in this study is still low compared with the study conducted in developed countries such as USA which has a self-reported screening uptake among HIV-positive women of about 75% [41]. This difference might be due to considerable attention has been given to cervical cancer prevention in developed countries compared with developing countries such as Ethiopia. This finding therefore highlights the need for focused interventions on the cervical cancer screening service uptake to ensure the effectiveness and to minimize cervical cancer mortality especially among high-risk groups like HIV-positive women.

We found ever screened women had a significantly higher perceived severity, higher perceived benefit, higher perceived self-efficacy, higher perceived threat and higher perceived net benefit than never screened women. This was consistent with the hypothesis of the Health Belief Model which states that, perceived severity and threat of cervical cancer, perceived benefit, perceived self-efficacy and net benefit about the preventive action of cervical cancer screening necessitates people to engage in preventive actions such as cervical cancer screening service uptake [26].

This study showed the odds of cervical cancer screening was about 6 and 3 times higher for those participants who worked as government employed (AOR 5.505 95% CI: 2.628, 11.532) and self-employed (AOR 3.047 95% CI: 1.298, 7.151) compared with unemployed. This is possibly because employed women have their own source of income, so they can consider their health issues as apriority.

In addition, employed women have more exposure to information and have different sources from which they can gather information unlike unemployed women. This finding is consistent with a study done in Malawiamong women who visited health centres [42].

A unit increase in the total score of perceived self-efficacy towards cervical cancer screening increased the odds of practicing cervical cancer screening by 24.20% (AOR 1.242, 95% CI: 1.128, 1.368). This highlights the importance of belief or confidence of a woman on her ability to successfully execute screening behavior to prevent herself from cervical cancer. This was supported by the Health Belief Model which stated that perceived self-efficacy is one of the predictors to affect the intended behavior and suggests that increasing women’s perceived self-efficacy has the potential to increase the likelihood of utilizing the service [26]. The finding was comparable with a systematic review conducted in Nigeria which revealed that confidence in one’s ability to make use of cervical cancer screening was responsible for women reported to have ever attended screening [43]. However,it was inconsistent with the study conducted in Florida, USA, HIV ambulatory clinics among HIV-positive women which showed that perceived self-efficacy was not significantly associated with CCS practice, [41] this variation might be due to the difference in sampling and socio-demographic characteristics.

The current study showed that a unit increase in the total score of perceived threat toward cervical cancer increased the odds of practicing cervical cancer screening by 8.60% (AOR 1.086, 95%CI: 1.052, 1.120). This might be explained by the assumption of the Health Belief Model, that a women is more likely to screen if she believes herself to be susceptible to cervical cancer and also considers the problem as serious, thus, their health actions were motivated in relation to the degree of threat [26]. Besides, this was due to the fact that the perceived threat lead HIV-positive women to perceive the impact of cervical cancer as devastating as HIV/AIDS, which might increasethe uptake of screening. Previous studies in both developing and developed countries showed the same finding the perception of susceptibility to cervical cancer and perception of seriousness of cervical cancer to predict cervical cancer screening behavior [21, 32, 41, 44, 45].

The other variable which was positively associated with cervical cancer screening practice was perceivednet benefit; a unit increase in total score of perceived net benefit increased the odds of practicing cervical cancer screening by 18.10% (AOR 1.181, 95%CI: 1.222, 1.243). This highlights the perceived benefit derived from cervical cancer screening among HIV positive women outweighs the perceived barriers which tends to hinder HIV-positive women’s ability to engage in cervical cancer screening service utilization.

Possibly this might be due to HIV-positive women having more contact with health care providers who were the main source of information about cervical cancer screening for the study participants, either due to regularly attending ART service or being prone to frequent hospitalization which in turn increased their perceived benefit towards screening. The finding goes in line with the concept of the Health Belief Model which stated that individuals are likely to utilize the screening service if they belief the benefit of being screened to prevent cervical cancer outweighs the cost of not being screened [26]. A study done in Botswana among women served by Mahalapye District Hospital also showed there was significant association between perceived benefits of screening and an uptake of the screening service [33].

Cues to action were not independent predictors for cervical cancer screening practice in the current study, implying that cues might enable HIV-positive women to have adequate information about screening. However, it does not necessarily mean cues influence screening behavior. For instance, it may enable them to know where to go for the test and what the test entails but irrespective of having cues for action other distal factors like acceptability of the service, quality of screening and treatment services mayaffect the real practice [26]. The finding was inconsistent with the study conducted in Ghana among HIV-positive women which reported cues about screening could improve cervical cancer screening practice and promote the health of high risk women [44]. This difference could be due to the difference in research design, theoretical basis that guided the studies and operationalization of concepts across the studies.

This study however should be interpreted in the light of its limitations. The study was based on participant self-report through interviews and the data collectors were health care providers of the study participants. These might be resulted in social desirability bias. Though we conducted the study in an ART clinic where a good number of HIV-positive women are accessed, we did not involve HIV-infected women who were not incare at the ART clinic who were also at increased risk for acquiring HPV and developing cervical cancer.

Perceived barrier construct of the HBM merely focused on the cognitive domain which fails to identify the actual barrier and also the HBM doesnot indicate clear operationalization instructions in linking perceived susceptibility and severity to threat and no formula was developed for overall behavioral evaluation [26]. These pitfalls of the model might have affected how the current study findings were generated.

## Conclusions

The findings of the current study have an important implication for public health intervention aimed at cervical cancer and its screening for HIV-positive women. The cervical cancer screening level in this study among HIV-positive women was lower than that of the recommended coverage of the target group by the national guideline and needs to be improved through creating awareness and educating HIV-positive women about the availability of screening and usefulness of utilizing the screening service.

The findings of this study suggested that perceived self-efficacy, perceived threat and perceived net-benefit were the predictors of cervical cancer screening practice. Educational programmes geared towards increasing perceived threat to cervical cancer, perceived self-efficacy and net-benefit toward screening can significantly improve the uptake of cervical cancer screening. During social and behavioural change communication material production, we suggest materials aimed at changing one’s perception to promote cervical cancer screening. We also recommend both quantitave and qualitative research and application of other behavioural models incorporating predictors other than cognitive related to overcome the limitation of this study.

## Supplementary information

**Additional file 1.** Interviewer administered structured questionnaire.

## Data Availability

The original raw data used in this study is available from the corresponding author and can be presented upon reasonable request.

## Abbrevations

AAPBCR: Addis Ababa Population Based Cancer Registry
ACS: American Cancer Society
AOR: Adjusted Odds Ratio
ART: Anti-Retroviral Treatment
AVE: Average variance extracted
BSc: Bachelor of Science
CC: Cervical Cancer
CCP: Cervical Cancer Programme
CCS: Cervical cancer screening
CFA: Confirmatory factor analysis
CHBMS: Champion’s revised Health Belief Model Scale
COR: Crude Odds Ratio
FMOH: Federal Ministry of Health
HBM: Health Belief Model
HIV/AIDS: Human Immune Deficiency Virus/ Acquired Immune Deficiency Syndrome
HIV: Human Immune Deficiency Virus
HPV: Human Papilloma Virus
KAP: Knowledge, Attitude, Practice
LMICs: Low and Middle Income Countries
NCDs: Non-communicable diseases
NGO: Non-Governmental Organization
REC: Research Ethical Committee
SBCC: Social and behavior change communication
SD: Standard-deviation
SPSS: Statistical Packages for Social Sciences
SVA: Single Visit Approach
TB: Tuberculosis
US$: United States’ dollar
VIA: Visual Inspection with Acetic Acid
WHO: World Health Organization
