# Supplementary material for: Predictors of cervical cancer screening practice among HIV positive women attending adult anti-retroviral treatment clinics in Bishoftu town, Ethiopia: the application of a health belief model
Source: BMC Cancer. 2019 Oct 23;19:989. doi: 10.1186/s12885-019-6171-6 (PMC6813043; doi:10.1186/s12885-019-6171-6)
Supplement: Supplementary file 1 — Additional file 1. Interviewer administered structured questionnaire. [file 12885_2019_6171_MOESM1_ESM.docx]

| **General information** | | | | | | | | | | | | | | | | | | | |
| --- | --- | --- | --- | --- | --- | --- | --- | --- | --- | --- | --- | --- | --- | --- | --- | --- | --- | --- | --- |
| **Questions** | | **Response & Coding Categories** | | | | | | | | | | | | | | | **Skip** | | |
| **Date of data collection** | | **dd/mm/yy** | | | | | | | | | | | | | | |  | | |
| **Code of data collectors** | |  | | | | | | | | | | | | | | |  | | |
| **Code of the facility** | |  | | | | | | | | | | | | | | |  | | |
| **Code of the questionnaire** | |  | | | | | | | | | | | | | | |  | | |
| **Total ART client in the facility** | |  | | | | | | | | | | | | | | |  | | |
| **Patient ID** | |  | | | | | | | | | | | | | | |  | | |
| **WHO clinical disease stage of the patient** | |  | | | | | | | | | | | | | | |  | | |
| **Patient CD4 count** | |  | | | | | | | | | | | | | | |  | | |
| **Section I Socio-demographic Characteristics** | | | | | | | | | | | | | | | | | | | |
| **SN** | **Questions** | **Response & Coding Categories** | | | | | | | | | | | | | | | **Skip** | | |
| **101** | **How old are you?(Complete in years)** | **……………………..** | | | | | | | | | | | | | | |  | | |
| **102** | **Where is your permanent residence?** | 1. **Bishoftu** 2. **Out of Bishoftu** | | | | | | | | | | | | | | |  | | |
| **103** | **What is your ethnicity?** | **1. Oromo**  **2. Amhara**  **3. Tigirie**  **4. Gurage**  **5. Other, specify………..** | | | | | | | | | | | | | | |  | | |
| **104** | **What is your current marital status** | **1. Single**  **2. Married**  **3. Divorced**  **4. Widowed** | | | | | | | | | | | | | | |  | | |
| **105** | **What is your religion** | **1. Orthodox**  **2. Muslim**  **3. Protestant**  **5. Other, Specify………….** | | | | | | | | | | | | | | |  | | |
| **106** | **What is your current educational Status** | **1. Unable to read and write**  **2. Able to read and write but not formally educated**  **3. If formally educated, what is the highest education level you have attained……………..** | | | | | | | | | | | | | | |  | | |
| **107** | **What is your current occupational status?** | **1. Government employee**  **2. Non-governmental employee**  **3. Merchant**  **4. Farmer**  **5. Daily laborer**  **6. Student**  **7.Unemployed**  **8. Other, specify …………** | | | | | | | | | | | | | | |  | | |
| **108** | **What is your monthly total house hold income?** | **…………..ETB** | | | | | | | | | | | | | | |  | | |
| **109** | **What is your husband educational status currently?** | **1. Illiterate**  **2. Read and write but no formal education**  **3. If formally educated, what is the last grade completed**  **……………..** | | | | | | | | | | | | | | |  | | |
| **110** | **What is your husband occupation currently?** | **1. Government employee**  **2. Non-governmental employee**  **3. Merchant**  **4. Farmer**  **5. Daily laborer**  **6. Student**  **7.Unemployed**  **8. Other, specify ……………** | | | | | | | | | | | | | | |  | | |
| **111** | **How many live births have you had?** | **1.One**  **2. Two**  **3.Three**  **4.More than three** | | | | | | | | | | | | | | |  | | |
| **112** | **When was your HIV diagnosis?** | **……………….(year or duration)** | | | | | | | | | | | | | | |  | | |
| **113** | **When was you started follow up in ART care……?** | **…………………(year or duration)** | | | | | | | | | | | | | | |  | | |
| **Section II Knowledge of the study participants on cervical cancer and cervical cancer screening** | | | | | | | | | | | | | | | | | | | |
| **SN** | **Questions** | | | **Response & Coding Categories** | | | | | | | | | | | | | **Skip** | | |
| **201** | **What is the cause of cervical cancer?** | | | 1. **Bacteria** 2. **Fungus** 3. **Virus** 4. **Hereditary/from family** 5. **I do not know** 6. **Other, specify…………………..** | | | | | | | | | | | | |  | | |
| **202** | **What are the symptoms of cervical cancer?** | | | 1. **Vaginal bleeding** 2. **Foul vaginal discharge** 3. **Pelvic or back pain** 4. **Post coital bleeding** 5. **I do not know** 6. **Other, specify……………….** | | | | | | | | | | | | |  | | |
| **203** | **What are the risk factors to cervical cancer?** | | | 1. **Age** 2. **Early onset of sexual intercourse** 3. **Having multiple sexual partners** 4. **Family history of cervical cancer** 5. **Cigarette smoking** 6. **I do not know** 7. **Other, specify………………..** | | | | | | | | | | | | |  | | |
| **204** | \| **Is cervical cancer preventable disease?** \| \| --- \| | | | 1. **Yes** 2. **No** 3. **I do not know** | | | | | | | | | | | | | **Go to Q.206** | | |
| **205** | **How can we prevent cervical cancer?** | | | 1. **Avoid multiple sexual partners** 2. **Avoid early onset sexual intercourse** 3. **Quit smoking** 4. **Through vaccination** 5. **Through screening services** 6. **Other, specify…………….** | | | | | | | | | | | | |  | | |
| **206** | **Is cervical cancer curable (treatable)?** | | | 1. **Yes** 2. **No** 3. **I do not know** | | | | | | | | | | | | | **Go to Q. 208** | | |
| **207** | **What things make cervical cancer curable once diagnosed?** | | | 1. **Seeking treatment at early stage** 2. **Seeking treatment at late stage** 3. **Seeking treatment at early or late stage** 4. **Other, specify………** | | | | | | | | | | | | |  | | |
| **208** | **Do you know any screening procedures to detect cervical cancer?** | | | 1. **Yes** 2. **No** 3. **I do not know** | | | | | | | | | | | | | **Go to Q.210** | | |
| **209** | **Which cervical cancer screening methods do you know?** | | | 1. **VIA** 2. **Pap smear** 3. **HPV test** 4. **Other, specify…………….** | | | | | | | | | | | | |  | | |
| **210** | **What is the aim of cervical cancer screening?** | | | 1. **To prevent cervical cancer** 2. **To early detection of cervical cancer** 3. **To treat cervical cancer** 4. **Other, specify……………..** | | | | | | | | | | | | |  | | |
| **211** | **When a woman should have screening?** | | | 1. **When menstruation starts** 2. **As soon as sexually active** 3. **At the age of 30** 4. **When starts having children** 5. **After menopause** 6. **Do not know** 7. **Other, specify…………..** | | | | | | | | | | | | |  | | |
| **212** | **When HIV positive woman should have screening?** | | | 1. **When menstruation starts** 2. **As soon as sexually active** 3. **At the age of 30** 4. **When start having children** 5. **After menopause** 6. **I do not know** 7. **Other, specify ……………** | | | | | | | | | | | | |  | | |
| **213** | \| **How frequent, screening should be done for cervical cancer?** \|  \| \| --- \| --- \| | | | \| 1. **Once every year** 2. **Once every three years** 3. **Once every 5 years** 4. **I do not know** 5. **Other, specify…….** \| \| --- \| | | | | | | | | | | | | |  | | |
| **214** | **How frequent, cervical cancer screening should be done for HIV positive women?** | | | 1. **Once every year** 2. **Once every two year** 3. **Once every three years** 4. **Once every 5 years** 5. **I do not know** 6. **Others specify………** | | | | | | | | | | | | |  | | |
| **Section III Study participants source of information about cervical cancer and cervical cancer screening** | | | | | | | | | | | | | | | | | | | |
| **SN** | **Questions** | **Response & Coding Categories** | | | | | | | | | | | | | | | **Skip** | | |
| **301** | **Have you ever heard about cervical cancer?** | 1. **Yes** 2. **No** | | | | | | | | | | | | | | | **Go to Q.401** | | |
| **302** | **From where did you hear about cervical cancer for the last time?** | 1. **Media (Television, Radio, Magazine, Brochures)** 2. **Health professional** 3. **School** 4. **Family** 5. **Friends** 6. **Other, specify……….** | | | | | | | | | | | | | | |  | | |
| **303** | **Have you ever heard about cervical cancer screening** | 1. **Yes** 2. **No** | | | | | | | | | | | | | | | **Go to Q.401** | | |
| **304** | **From where did you heard about cervical cancer screening methods for the last time?** | \| 1. **Media** 2. **Health professionals** 3. **School** 4. **Family** 5. **Friends** 6. **Other, specify……** \| \| --- \| | | | | | | | | | | | | | | |  | | |
| **Section IV Cervical cancer screening practice** | | | | | | | | | | | | | | | | | | | |
| **SN** | **Questions** | **Response & Coding Categories** | | | | | | | | | | | | | | | **Skip** | | |
| **401** | **Have you ever had cervical cancer screening?** | 1. **Yes** 2. **No** | | | | | | | | | | | | | | | **Go to Q.406** | | |
| **402** | **Why did you screen?** | 1. **Health professional’s advise/ recommendation** 2. **Media(Television/ radio/ magazines/ brochures)** 3. **Relative/ friend recommendation** 4. **It’s a standard/routine care at clinic** 5. **Other, specify……….** | | | | | | | | | | | | | | |  | | |
| **403** | **Have you screened for cervical cancer after you were diagnosed with HIV/AIDS?** | 1. **Yes** 2. **No** | | | | | | | | | | | | | | | **Go to Q.405** | | |
| **404** | **How many times have you screened after you were diagnosed with HIV/AIDS?** | **……………………** | | | | | | | | | | | | | | |  | | |
| **405** | **When was the last time you screened for cervical cancer?** | 1. **within the past three years** 2. **within the past five years** 3. **More than five years** 4. **Other, Specify…….** | | | | | | | | | | | | | | |  | | |
| **406** | **Why did not you screen** | 1. **It is expensive** 2. **Never to think of it** 3. **It is painful** 4. **I am healthy** 5. **Do not know about it** 6. **Partner attitude** 7. **Fear of positive result** 8. **Religion factors** 9. **Other, Specify………….** | | | | | | | | | | | | | | |  | | |
| **V. Perceived susceptibility to cervical cancer** | | | | | | | | | | | | | | | | | | | |
| **SN** | **Questions** | | **Response and coding categories** | | | | | | | | | | | | | | | | |
|  |  | | **Strongly disagreed** | | | | **Disagree** | | | **Not-sure/**  **don’t know** | | | | **Agree** | | | | | **Strongly agree** |
| **501** | **It is likely that I will get cervical cancer in the future** | | **1** | | | | **2** | | | **3** | | | | **4** | | | | | **5** |
| **502** | **Older women are more at risk of cervical cancer than younger women** | | **1** | | | | **2** | | | **3** | | | | **4** | | | | | **5** |
| **503** | **Every woman of child bearing age is at risk of cervical cancer** | | **1** | | | | **2** | | | **3** | | | | **4** | | | | | **5** |
| **504** | **Cervical cancer is more common to women who are HIV positive** | | **1** | | | | **2** | | | **3** | | | | **4** | | | | | **5** |
| **505** | **I think that women with multiple sexual partners are more prone to cervical cancer** | | **1** | | | | **2** | | | **3** | | | | **4** | | | | | **5** |
| **vi. perceived seriousness(severity) to cervical cancer** | | | | | | | | | | | | | | | | | | | |
| **SN** | **Questions** | | | | | **Response and coding categories** | | | | | | | | | | | | | |
|  |  | | | | | **Strongly disagree** | | | **Disagree** | | | **Not-sure/**  **I don’t know** | | | | **Agree** | | **Strongly agree** | |
| **601** | **The thought of cervical cancer scares me** | | | | | **1** | | | **2** | | | **3** | | | | **4** | | **5** | |
| **602** | **When I think about cervical cancer, my heart beats faster** | | | | | **1** | | | **2** | | | **3** | | | | **4** | | **5** | |
| **603** | **Cervical cancer is not as serious as other types of cancers** | | | | | **1** | | | **2** | | | **3** | | | | **4** | | **5** | |
| **604** | **If I got cervical cancer, it would be more serious than HIV negative’s women** | | | | | **1** | | | **2** | | | **3** | | | | **4** | | **5** | |
| **605** | **I feel I would not live longer five years if I got cervical cancer** | | | | | **1** | | | **2** | | | **3** | | | | **4** | | **5** | |
| **606** | **I believe that cervical cancer can be cured easily** | | | | | **1** | | | **2** | | | **3** | | | | **4** | | **5** | |
| **607** | **If I had cervical cancer my whole life would change** | | | | | **1** | | | **2** | | | **3** | | | | **4** | | **5** | |
| **608** | **Death resulting from cervical cancer is rare** | | | | | **1** | | | **2** | | | **3** | | | | **4** | | **5** | |
| **609** | **Cervical cancer would threaten a relationship**  **with my husband or partner** | | | | | **1** | | | **2** | | | **3** | | | | **4** | | **5** | |
| **VII. Perceived benefits of doing of cervical cancer screening** | | | | | | | | | | | | | | | | | | | |
| **SN** | **Questions** | | | | | **Response and coding categories** | | | | | | | | | | | | | |
|  |  | | | | | **Strongly disagree** | | | **Disagree** | | | **Not-sure/**  **I don’t know** | | | | **Agree** | | **Strongly agree** | |
| **701** | **I think that having early cervical cancer screening will decrease complications of cervical cancer** | | | | | **1** | | | **2** | | | **3** | | | | **4** | | **5** | |
| **702** | **Having regular cervical cancer screening will help to find changes in the cervix before they turn into cancer** | | | | | **1** | | | **2** | | | **3** | | | | **4** | | **5** | |
| **703** | **If cervical changes are found early from cervical cancer screening, they are easily curable.** | | | | | **1** | | | **2** | | | **3** | | | | **4** | | **5** | |
| **704** | **I think that cervical cancer screening can reduce the chance of having advanced cervical cancer in HIV positive women** | | | | | **1** | | | **2** | | | **3** | | | | **4** | | **5** | |
| **705** | **Cervical cancer screening can decrease cervical cancer associated death in HIV positive women** | | | | | **1** | | | **2** | | | **3** | | | | **4** | | **5** | |
| **706** | **I would not be so anxious about cervical cancer if I got regular screening** | | | | | **1** | | | **2** | | | **3** | | | | **4** | | **5** | |
| **VIII. Perceived barriers to cervical cancer screening** | | | | | | | | | | | | | | | | | | | |
| **SN** | **Questions** | | | | **Response and coding categories** | | | | | | | | | | | | | | |
|  |  | | | | **Strongly disagree** | | | **Disagree** | | | **Not-sure/ I don’t know** | | | | **Agree** | | | **Strongly agree** | |
| **801** | **Cervical cancer screening is embarrassing to me** | | | | **1** | | | **2** | | | **3** | | | | **4** | | | **5** | |
| **802** | **Having cervical cancer screening is too painful** | | | | **1** | | | **2** | | | **3** | | | | **4** | | | **5** | |
| **803** | **If a young unmarried woman does cervical cancer screening, everyone will think that she is having sex** | | | | **1** | | | **2** | | | **3** | | | | **4** | | | **5** | |
| **804** | **Getting cervical cancer screening increase anxiety about liability of having cervical cancer** | | | | **1** | | | **2** | | | **3** | | | | **4** | | | **5** | |
| **805** | **If there is cervical cancer development in my destiny, having cervical cancer screening cannot prevent it** | | | | **1** | | | **2** | | | **3** | | | | **4** | | | **5** | |
| **806** | **If a woman has not had sex, cervical cancer screening will take away her virginity** | | | | **1** | | | **2** | | | **3** | | | | **4** | | | **5** | |
| **807** | **Only women who have had children need to do cervical cancer screening.** | | | | **1** | | | **2** | | | **3** | | | | **4** | | | **5** | |
| **808** | **My husband or partner will not allow me to do cervical cancer screening** | | | | **1** | | | **2** | | | **3** | | | | **4** | | | **5** | |
| **809** | **Lack of female screeners in health facilities will discourage women from having cervical cancer screening** | | | | **1** | | | **2** | | | **3** | | | | **4** | | | **5** | |
| **810** | **Attitudes/care of health workers can discourage from going for cervical cancer screening.** | | | | **1** | | | **2** | | | **3** | | | | **4** | | | **5** | |
| **811** | **There is no health center close to my house to have cervical cancer screening** | | | | **1** | | | **2** | | | **3** | | | | **4** | | | **5** | |
| **812** | **Having cervical cancer screening takes too much time** | | | | **1** | | | **2** | | | **3** | | | | **4** | | | **5** | |
| **813** | **I will never have cervical cancer screening if I have to pay for it** | | | | **1** | | | **2** | | | **3** | | | | **4** | | | **5** | |
| **814** | **I would be ashamed to show my private parts to have cervical cancer screening** | | | | **1** | | | **2** | | | **3** | | | | **4** | | | **5** | |
| **815** | **I am afraid to expose my private parts to have cervical cancer screening because is not acceptable by my religion** | | | | **1** | | | **2** | | | **3** | | | | **4** | | | **5** | |
| **IX. Perceived self-efficacy to practice cervical cancer screening** | | | | | | | | | | | | | | | | | | | |
| **SN** | **Questions** | | | | | **Response and coding categories** | | | | | | | | | | | | | |
|  |  | | | | | **Strongly disagree** | | | **Disagree** | | | | **Not-sure/**  **don’t know** | | | **Agree** | | **Strongly agree** | |
| **901** | **I know where to get the service of cervical cancer screening** | | | | | **1** | | | **2** | | | | **3** | | | **4** | | **5** | |
| **902** | **I am confident that I can schedule regular appointment for cervical cancer screening** | | | | | **1** | | | **2** | | | | **3** | | | **4** | | **5** | |
| **903** | **I can talk to people in my life to have cervical cancer screening** | | | | | **1** | | | **2** | | | | **3** | | | **4** | | **5** | |
| **904** | **I can find a way to pay for having cervical cancer screening** | | | | | **1** | | | **2** | | | | **3** | | | **4** | | **5** | |
| **905** | **I am sure of that I can go to health facility to get screened if I want to get it** | | | | | **1** | | | **2** | | | | **3** | | | **4** | | **5** | |
| **X. Cues for action** | | | | | | | | | | | | | | | | | | | |
| **SN** | **Questions** | | | | | **Response and coding categories** | | | | | | | | | | | | **Skip** | |
| **1001** | **Have you ever known women who**  **Screened to cervical cancer?** | | | | | 1. **No** 2. **Yes** | | | | | | | | | | | |  | |
| **1002** | **Do you have family history of cervical cancer?** | | | | | 1. **No** 2. **Yes** | | | | | | | | | | | |  | |
| **1003** | **Have you ever heard through media or read about cervical cancer ?** | | | | | 1. **No** 2. **Yes** | | | | | | | | | | | |  | |
| **1004** | **Has your physician ever talked to you about cervical cancer screening?** | | | | | 1. **No** 2. **Yes** | | | | | | | | | | | |  | |
